# Consistency and correlation of a domestic Kehua serum free light chain assay with two imported analytical systems

**DOI:** 10.3389/fonc.2026.1900671

**Published:** 2026-10-15

**Authors:** Yansong Ren, Shoulei Wang, Limin Han, Yansheng Wang, Qian Ren, Mengxin Wang, Yonghui Xia, Guoqing Zhu

**Affiliations:** 1 State Key Laboratory of Experimental Hematology, National Clinical Research Center for Blood Diseases, Haihe Laboratory of Cell Ecosystem, Institute of Hematology & Blood Diseases Hospital, Chinese Academy of Medical Sciences & Peking Union Medical College, Tianjin, China; 2 Shanghai Kehua Bio-Engineering Co., Ltd, Reagent R and D Center, Shanghai, China

**Keywords:** immunoassay, immunoglobulin light chains, multiple myeloma, paraproteinemias, quality control

## Abstract

**Background:**

Serum free light chain (sFLC) testing is central to the diagnosis and monitoring of plasma cell disorders, but assay availability and implementation can vary by laboratory setting. We evaluated a domestic latex-enhanced immunoturbidimetric Kehua serum sFLC assay against Freelite and N Latex systems.

**Methods:**

Residual serum samples from 133 adults with suspected or established monoclonal gammopathies were measured in parallel by Kehua and Freelite; 122 were also tested by N Latex. Precision was assessed with low- and high-level serum controls. Method agreement was evaluated using correlation, Passing-Bablok regression, Bland-Altman analysis, qualitative concordance and comparator-defined ROC analysis.

**Results:**

Kehua within-run CVs were <5% for κ and λ controls. Agreement with Freelite was excellent for FLC-κ, FLC-λ and κ/λ ratio (Pearson r = 0.988-1.000; CCC = 0.983-0.999), with Passing-Bablok slopes close to unity. Agreement with N Latex was also strong (r = 0.991-0.998), although κ/λ ratio showed proportional bias. Total qualitative concordance was 89.47-93.23% versus Freelite and 83.61-91.80% versus N Latex, with Cohen’s κ of 0.67-0.85. Comparator-defined ROC AUCs were 0.994-0.999 for elevated FLC-κ or FLC-λ and 0.796-0.869 for κ/λ ratio abnormality.

**Conclusion:**

The Kehua serum assay demonstrated acceptable precision and good clinical agreement with two imported methods. It may serve as a domestic option for routine serum sFLC quantification, provided method-specific intervals are maintained and the same platform is used for serial monitoring.

## 1 Introduction

Multiple myeloma (MM) is the second most common haematological malignancy worldwide and is characterized by the clonal proliferation of bone marrow plasma cells secreting a monoclonal immunoglobulin or its fragments (1, 2). The age-standardized incidence of MM has risen substantially over the past three decades, driven mainly by population ageing and improved diagnostic awareness. In China the burden has shown a particularly steep upward trajectory, with provincial age-standardized incidence rates expected to continue rising through 2050 (3, 4). Beyond MM, the spectrum of plasma cell disorders includes monoclonal gammopathy of undetermined significance (MGUS), smouldering myeloma, light-chain amyloidosis (AL) and rarer entities such as light-chain deposition disease and POEMS syndrome (1, 5).

Serum free light chain (sFLC) measurement has become a cornerstone laboratory test for these disorders. The current International Myeloma Working Group (IMWG) criteria explicitly include an involved/uninvolved sFLC ratio ≥ 100 as a myeloma-defining biomarker, provided the absolute concentration of the involved light chain is ≥ 100 mg/L (1, 2). The sFLC assay is now recommended together with serum protein electrophoresis and immunofixation for screening, and is the preferred quantitative marker for monitoring oligosecretory and non-secretory myeloma and AL amyloidosis (6). For high-risk smouldering MM, sFLC ratio cut-offs of 20 and 100 are used in the contemporary 2/20/20 risk-stratification model (2, 7).

Two commercial nephelometric or turbidimetric sFLC assays have dominated the field. The Freelite assay (The Binding Site, Birmingham, UK), launched in 2001, uses sheep polyclonal antibodies directed against hidden epitopes of light chains and is regarded as the IMWG reference (6, 8). The N Latex FLC assay (Siemens Healthineers Diagnostics, Marburg, Germany) uses a mixture of monoclonal antibodies and was introduced as an alternative reagent on the Siemens BN system. Multiple head-to-head studies have demonstrated that, although both assays correlate well, they are not interchangeable: Freelite tends to overestimate FLC-κ and κ/λ ratio, whereas N Latex tends to give higher FLC-λ values, and clinically relevant discrepancies can occur in up to 5% of patients (8–11). Even within the IMWG community there is ongoing debate as to whether decision thresholds derived from Freelite can be cross-applied to N Latex (11, 12).

The technical limitations common to both imported assays include batch-to-batch variability, antigen-excess (prozone) effects, non-linearity after dilution and the requirement for dedicated high-cost nephelometric platforms (13, 14). The reagents are also imported, which may affect supply continuity and local implementation in some settings, although this study did not collect formal cost, distribution or laboratory-implementation data. Domestic immunoturbidimetric reagents are generally latex-enhanced particle immunoassays: anti-human free κ or free λ antibodies are immobilized on latex particles, and antigen-antibody complex formation increases turbidity that can be quantified on routine automated chemistry analysers. Depending on the manufacturer, such reagents may use monoclonal, polyclonal, mixed or otherwise proprietary antibody preparations; therefore, antibody source and clonality should be reported whenever available. In principle, this format can allow sFLC testing to be incorporated into laboratories that already operate automated biochemistry platforms, but clinical adoption requires method-comparison and full analytical validation rather than assumptions based on origin or platform alone (15).

The Kehua FLC assay (Shanghai Kehua Bio-engineering Co., Ltd., Shanghai, China) is one such domestic latex-enhanced immunoturbidimetric reagent designed to run on routine biochemistry platforms such as the Beckman Coulter AU series. Before such an assay can be integrated into routine workflow it must be shown to meet established performance standards (16, 17) and to provide results that are sufficiently consistent with the established imported assays in terms of both quantitative concentrations and qualitative classification. The objective of the present study was therefore to evaluate the analytical precision, the quantitative correlation and the qualitative agreement of the Kehua serum sFLC assay against Freelite and N Latex in a cohort of patients with suspected or established plasma cell disorders, and to assess its comparator-defined classification performance using the imported methods as reference comparators rather than as an independent disease gold standard.

## 2 Materials and methods

### 2.1 Study population and sample collection

This was a single-centre, prospective method-comparison study conducted at the Department of Hematology of the Institute of Hematology & Blood Diseases Hospital, between 19 August and 15 October 2025. Consecutive residual serum samples submitted for sFLC testing were enrolled when there was sufficient remaining volume for parallel analysis on all three measurement systems. Inclusion criteria were as follows: (1) age ≥ 18 years; (2) availability of at least 500 µL of serum after the routine workup had been completed; (3) full clinical information available, including a working diagnosis of multiple myeloma, MGUS, AL amyloidosis, smouldering myeloma, Waldenström macroglobulinemia or evaluation of suspected monoclonal gammopathy. Samples were excluded if they were grossly haemolysed, lipaemic or icteric, or if they had been stored for more than 7 days at 2-8 °C before testing. The clinical spectrum of the enrolled serum samples included IgG-κ, IgG-λ, IgA-κ, IgA-λ, IgD-λ, free light-chain only myeloma, Waldenström macroglobulinemia, AL amyloidosis and MGUS. A total of 133 serum samples were tested on the Kehua and Freelite systems and 122 of these were additionally tested on the Siemens N Latex system after exclusion of samples with insufficient remaining volume for the third measurement. Urine and cerebrospinal fluid specimens were not included. The study protocol followed the principles of the Declaration of Helsinki, and the use of residual de-identified samples was approved by the institutional ethics committee.

### 2.2 Instruments and reagents

Three independent sFLC measurement systems were used in parallel, each operated according to the manufacturer’s instructions and under routine quality-control programmes. The first was the Kehua FLC test (kappa and lambda separately), comprising a latex-enhanced immunoturbidimetric reagent (Shanghai Kehua Bio-engineering Co., Ltd., Shanghai, China; FLC-κ reagent lot 2024125Z, expiry 9 December 2025; FLC-λ reagent lot 202412120, expiry 10 December 2025), calibrated with manufacturer-assigned in-house calibrators. According to the product information available for the evaluated reagent lots, the Kehua assay uses anti-human free κ and anti-human free λ antibodies immobilized on latex particles; however, the manufacturer did not disclose whether these antibodies are monoclonal, polyclonal, recombinant or mixed preparations, nor the detailed production route. Because there is no universally accepted international reference material for serum free light chains, the Kehua calibrators were traceable to the manufacturer’s internal master-calibration system rather than to an external reference standard. The Kehua reagent was operated on a Beckman Coulter AU5800 fully automated chemistry analyser (Beckman Coulter, Brea, CA, USA). The second was the Freelite assay (The Binding Site, Birmingham, UK), a polyclonal sheep-antibody-based particle-enhanced turbidimetric assay, which was also run on the Beckman Coulter AU5800 in this study. The third was the N Latex FLC assay (Siemens Healthineers Diagnostics GmbH, Marburg, Germany; reagent lots 473192 [κ, expiry 15 January 2026] and 473202 [λ, expiry 1 April 2026]), a particle-enhanced nephelometric assay using a defined mixture of monoclonal antibodies and run on a BN II System nephelometer.

The manufacturer-specified reference intervals were as follows: Freelite κ 3.3-19.4 mg/L, λ 5.7-26.3 mg/L and κ/λ ratio 0.26-1.65; N Latex κ 6.7-22.4 mg/L, λ 8.3-27.0 mg/L and κ/λ ratio 0.31-1.56 (9, 10). The Kehua assay was operated using the same range categories as its corresponding comparator for each pairwise analysis (i.e., Freelite intervals for Kehua-versus-Freelite, N Latex intervals for Kehua-versus-N Latex), in keeping with the recommendation that each method retains its own decision thresholds.

### 2.3 Experimental procedure and quality control

All measurements were performed on the same serum specimen within 24 h of collection. Each sample was processed independently on the three platforms with operators blinded to the results of the other systems. The following workflow was applied to every sample: (1) centrifugation at 3 000 × g for 10 min within 2 h of phlebotomy; (2) primary measurement at the manufacturer-recommended initial dilution; (3) *post-hoc* dilution and reanalysis when the primary value exceeded the upper limit of linearity, applying dilution factors of 1:10, 1:50, 1:100 or higher as required and recovery checked against a second dilution; (4) repeated testing in duplicate when antigen excess (hook effect) was suspected on the basis of a non-linear post-dilution recovery; (5) recording of qualitative classification (positive or negative) for κ, λ and κ/λ ratio using the system-specific reference intervals.

Internal quality control was performed on every analytical run using two levels of commercial quality-control materials provided by Kehua (κ low/high lots 202506120 and 473685; λ controls operated against the same lot of platform reagents), and equivalent low and high controls were used for Freelite and N Latex. Within-run precision was assessed by analysing each control material five times consecutively on the same day, calculating the mean, standard deviation (SD) and coefficient of variation (CV) for each level. Acceptance criteria followed the Clinical and Laboratory Standards Institute (CLSI) EP05-A3 guideline (18), requiring CV ≤ 10% at both control levels and means falling within the manufacturer-specified ranges. The QC records available for the submitted dataset included four independent FLC-κ run dates (22 August, 6 September, 22 September and 18 October 2025) and two independent FLC-λ run dates (22 September and 18 October 2025), thereby documenting within-run performance across the reagent lots used during the recruitment period while avoiding overstatement of a full EP05 precision design.

### 2.4 Statistical analysis

All statistical analyses were performed in Python 3.11 (using the SciPy, NumPy, scikit-learn and Matplotlib packages) and MedCalc v20.0 (MedCalc Software Ltd., Ostend, Belgium). Continuous variables are presented as median and interquartile range because the FLC distributions were strongly right-skewed. The strength of association between methods was quantified using Pearson’s correlation coefficient (after log transformation when necessary), Spearman’s rank correlation coefficient and Lin’s concordance correlation coefficient (CCC), the latter combining precision and accuracy into a single index. Following the recommendations of CLSI EP09c (16, 19) and Bilic-Zulle (20), systematic and proportional bias between methods were estimated by Passing-Bablok regression, which is robust to non-normal residuals and outliers, yielding slope and intercept point estimates. Agreement was further assessed by Bland-Altman analysis of relative percentage differences plotted against the geometric mean to accommodate the wide dynamic range of the assay (21). The mean relative difference and the 95% limits of agreement (LOA, defined as mean ± 1.96 SD) were computed.

For qualitative classification, contingency tables were constructed for each method pair using the manufacturer-specific reference intervals, and the following metrics were calculated: (1) positive concordance rate (PPV-style agreement), (2) negative concordance rate, (3) overall concordance rate and (4) Cohen’s unweighted kappa coefficient with its 95% confidence interval; kappa values were interpreted following Landis and Koch as poor (< 0.20), fair (0.21-0.40), moderate (0.41-0.60), substantial (0.61-0.80) or almost-perfect (> 0.80). Comparator-defined classification performance of the Kehua assay was further evaluated by receiver-operating characteristic (ROC) analysis, treating the imported method’s qualitative classification as the reference comparator. For FLC-κ and FLC-λ the analysis used the raw Kehua concentration as the predictor against the imported method’s call of “value above the upper reference limit”; for the κ/λ ratio the analysis used the absolute value of log_10_(κ/λ) as the predictor of κ/λ abnormality (i.e., outside the reference interval in either direction). The area under the curve (AUC), the optimal Youden index threshold and the corresponding sensitivity and specificity were reported as measures of concordance with comparator classification, not as diagnostic accuracy against an independent clinical gold standard. A two-sided P-value < 0.05 was considered statistically significant.

## 3 Results

### 3.1 Quality-control performance of the Kehua FLC assay

The available internal quality-control results for the Kehua FLC-κ and FLC-λ reagents are summarized in **Table 1**. For FLC-κ, four independent run dates were available. The mean values at the low- and high-concentration controls were 12.07-12.52 mg/L and 51.34-52.41 mg/L, respectively, all of which fell well within the manufacturer-stated acceptance ranges of 10.2-13.8 mg/L and 42.3-57.2 mg/L. Within-run CV values for FLC-κ ranged from 1.52% to 3.88% at the low control and from 0.95% to 2.67% at the high control, with all eight individual CV measurements satisfying the < 10% precision goal recommended by CLSI EP05-A3 (18). For FLC-λ, two independent run dates were available. Mean low-control and high-control results were 24.30-24.38 mg/L and 58.32-60.02 mg/L (target ranges 21.7-29.4 mg/L and 51.6-69.8 mg/L), with within-run CV values of 2.43-3.58% at the low level and 0.67-4.66% at the high level. Taken together, the available QC dataset demonstrated acceptable within-run precision across both reagent lots used during the study period, although it should not be interpreted as a complete long-term EP05 precision study.

**Table 1:** Internal quality-control performance of the Kehua FLC-κ reagent across four independent analytical runs and the Kehua FLC-λ reagent across two independent analytical runs on the AU5800 analyser.

Analyte	Run date	Level	Mean (mg/L)	SD	CV (%)	Target (mg/L)	Acceptable range (mg/L)
FLC-κ	2025-08-22	Low	12.07	0.35	2.91	12.0	10.2-13.8
FLC-κ	2025-08-22	High	51.34	1.37	2.67	49.8	42.3-57.2
FLC-κ	2025-09-06	Low	12.25	0.21	1.75	12.0	10.2-13.8
FLC-κ	2025-09-06	High	52.41	0.50	0.95	49.8	42.3-57.2
FLC-κ	2025-09-22	Low	12.44	0.48	3.88	12.0	10.2-13.8
FLC-κ	2025-09-22	High	52.24	1.16	2.22	49.8	42.3-57.2
FLC-κ	2025-10-18	Low	12.52	0.19	1.52	12.0	10.2-13.8
FLC-κ	2025-10-18	High	52.40	1.30	2.48	49.8	42.3-57.2
FLC-λ	2025-09-22	Low	24.30	0.59	2.43	25.6	21.7-29.4
FLC-λ	2025-09-22	High	60.02	2.80	4.66	60.7	51.6-69.8
FLC-λ	2025-10-18	Low	24.38	0.87	3.58	25.6	21.7-29.4
FLC-λ	2025-10-18	High	58.32	0.39	0.67	60.7	51.6-69.8

SD, standard deviation; CV, coefficient of variation; each within-run estimate based on n = 5 replicates per control level. Manufacturer’s target and acceptable ranges are taken from the reagent insert. FLC-κ controls were available for four run dates, whereas FLC-λ controls were available for two run dates in the submitted QC dataset.

### 3.2 Quantitative correlation of FLC-κ and FLC-λ between systems

The 133 samples covered a wide concentration spectrum, with Kehua FLC-κ ranging from 0.72 mg/L to 237.91 mg/L (median 12.58 mg/L) and Kehua FLC-λ ranging from 2.00 mg/L to 9 529.02 mg/L (median 9.92 mg/L). The distribution of values across the three systems is illustrated by the combined box-and-violin plots in **Figure 1**, which shows that the central tendencies and the overall shapes of the FLC-κ, FLC-λ and κ/λ ratio distributions were very similar between the Kehua, Freelite and N Latex platforms over five orders of magnitude (log scale).

**Figure 1:**
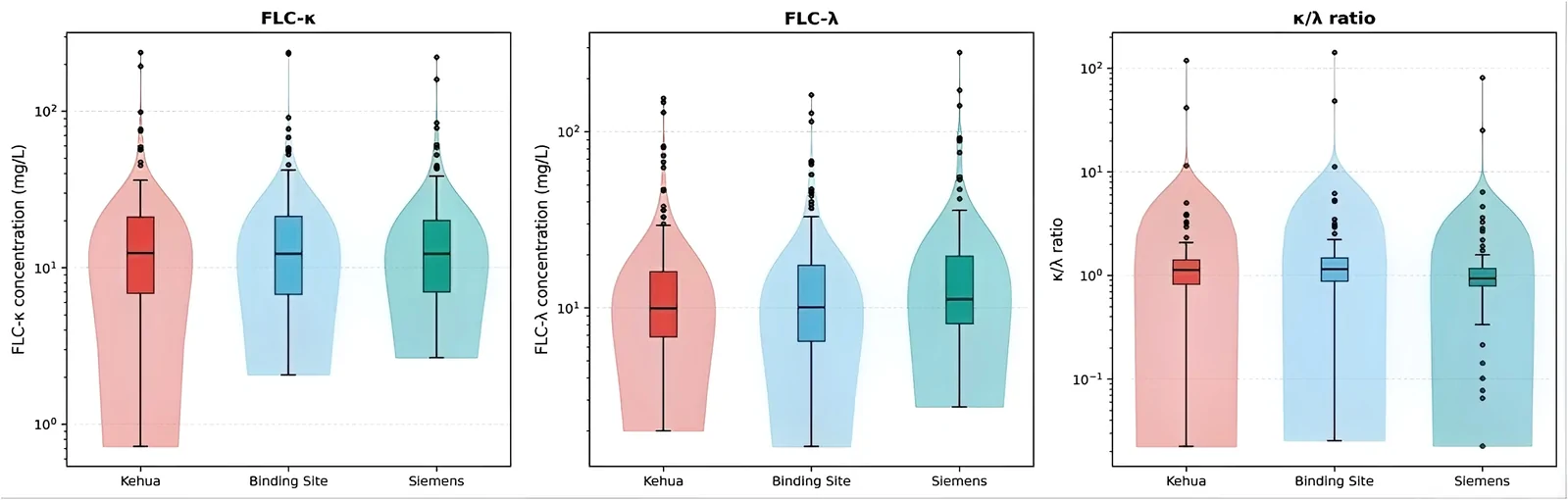
Distribution of FLC-κ, FLC-λ and κ/λ ratio measured by the Kehua, Freelite and N Latex assays. Box plots show the median and interquartile range; violins display the underlying probability density. The y-axes are on a logarithmic scale. Only serum samples were analysed. Samples with FLC-λ > 1–000 mg/L were excluded from the display only for clarity but were retained in all statistical analyses.

Pairwise quantitative agreement between the Kehua assay and the two imported methods is summarized in **Table 2**. Pearson correlation coefficients for FLC-κ and FLC-λ exceeded 0.99 for both comparisons. Lin’s CCC was 0.986 (Kehua-Freelite, FLC-κ) and 0.984 (Kehua-N Latex, FLC-κ), confirming both high precision and high accuracy of agreement. Passing-Bablok regression demonstrated near-identity for Kehua-Freelite κ measurement (slope = 0.989, intercept = 0.223 mg/L), a modest positive proportional bias for Kehua-Freelite λ (slope = 1.077, intercept = −0.787 mg/L), a small negative proportional bias for Kehua-N Latex κ (slope = 0.870, intercept = 1.063 mg/L), and a positive proportional bias for Kehua-N Latex λ (slope = 1.138, intercept = 0.230 mg/L) (**Figure 2**). On a log-log scale all six regression lines tracked the line of identity closely across the entire reportable range.

**Table 2:** Quantitative agreement between the Kehua assay and the two imported reference systems, including Pearson correlation, Spearman correlation, Lin’s concordance correlation coefficient and Passing-Bablok regression.

Comparison	Analyte	n	Pearson r	Spearman r	Lin’s CCC	PB slope	PB intercept
Kehua vs Freelite	FLC-κ	133	0.988	0.974	0.986	0.989	0.223
Kehua vs Freelite	FLC-λ	133	1.000	0.951	0.999	1.077	−0.787
Kehua vs Freelite	κ/λ ratio	133	0.999	0.825	0.983	1.022	0.018
Kehua vs N Latex	FLC-κ	122	0.991	0.978	0.984	0.870	1.063
Kehua vs N Latex	FLC-λ	122	0.995	0.953	0.569	1.138	0.230
Kehua vs N Latex	κ/λ ratio	122	0.998	0.834	0.922	0.733	0.123

CCC, concordance correlation coefficient; PB, Passing-Bablok regression. All P-values for Pearson correlation < 0.001. The reduced CCC for Kehua vs N Latex FLC-λ reflects the influence of a single extreme outlier with FLC-λ > 9–000 mg/L; Pearson correlation, which is on a different scale, remained excellent.

**Figure 2:**
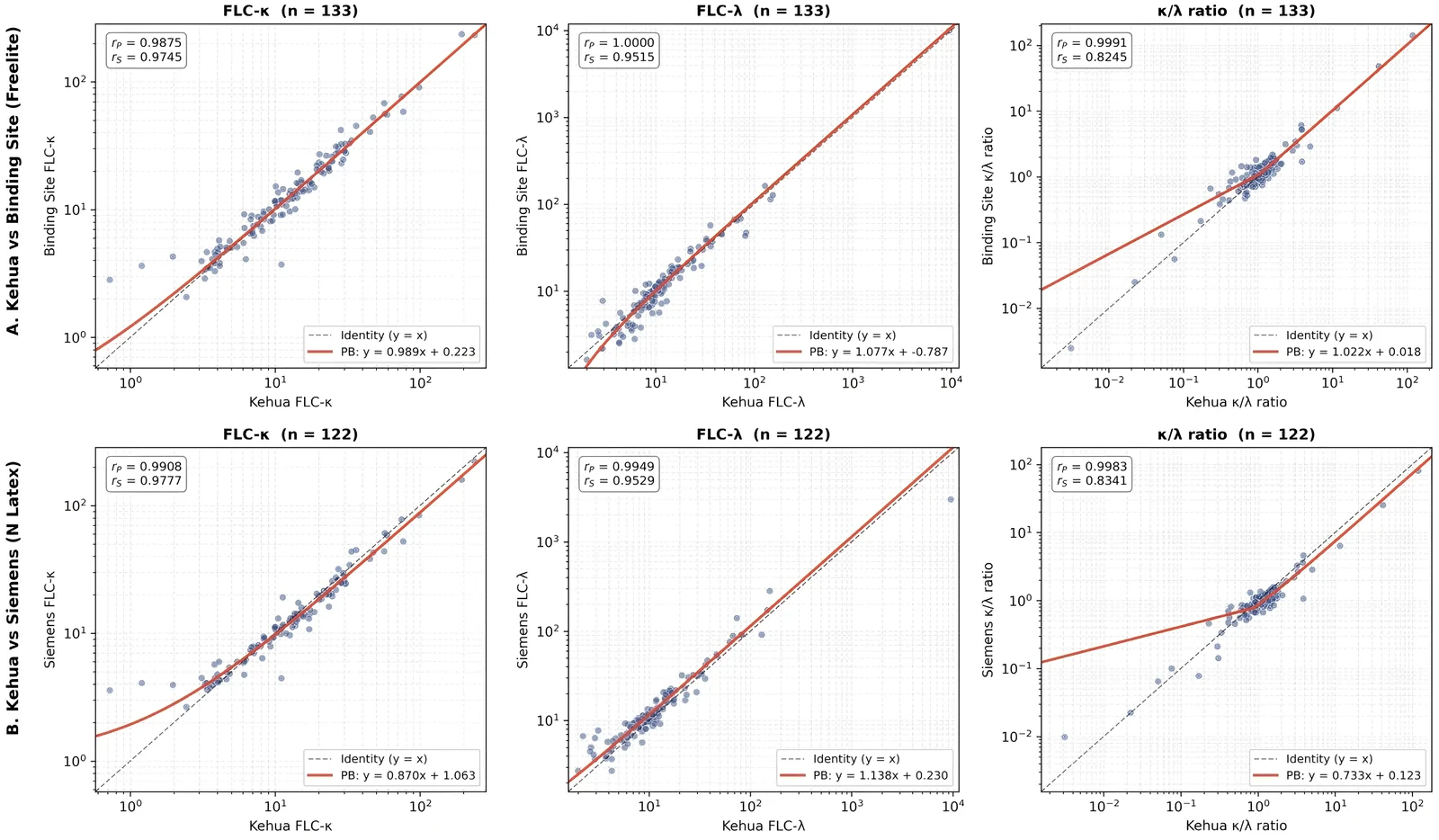
Scatter plots and Passing-Bablok regression lines (red) compared with the identity line (dashed black) for the six pairwise comparisons of Kehua against the two imported assays. Pearson (rP) and Spearman (rS) correlation coefficients are annotated in each panel. Both axes are logarithmic.

### 3.3 Correlation and agreement of the κ/λ ratio

Quantitative agreement was particularly strong for the κ/λ ratio against the Freelite assay (Pearson r = 0.999, Passing-Bablok slope 1.022, intercept 0.018; **Table 2**), indicating that the diagnostically critical ratio was closely aligned between Kehua and Freelite over a κ/λ range spanning from 0.003 to 119. Against N Latex the κ/λ ratio also correlated strongly (Pearson r = 0.998), but the Passing-Bablok slope of 0.733 indicates a clinically relevant proportional bias such that, at high κ/λ values, the Kehua-derived ratio underestimates the N Latex value, whereas at low ratios it overestimates it. This pattern is visible in the lower right panel of **Figure 2** and means that absolute κ/λ ratio values from Kehua and N Latex should not be used interchangeably around ratio-based decision points.

Bland-Altman analysis of relative differences is shown in **Figure 3**. For the Kehua-Freelite comparison the mean relative difference (bias) was −3.75% for FLC-κ, +3.63% for FLC-λ and −7.34% for the κ/λ ratio, with all three Bland-Altman plots showing the differences scattered symmetrically around the mean line across more than two orders of magnitude of mean concentration. The 95% limits of agreement were [−48.2%, +40.7%] for FLC-κ, [−45.7%, +52.9%] for FLC-λ and [−64.2%, +49.5%] for the κ/λ ratio. For the Kehua-N Latex comparison the mean relative differences were −0.16%, −13.05% and +12.56% for FLC-κ, FLC-λ and the κ/λ ratio, respectively, with 95% LOA of [−47.5%, +47.2%], [−63.3%, +37.2%] and [−44.7%, +69.8%]. Of note, the Bland-Altman plots showed no obvious concentration-dependent trend in either comparison, supporting the use of a constant relative-difference model rather than a proportional one.

**Figure 3:**
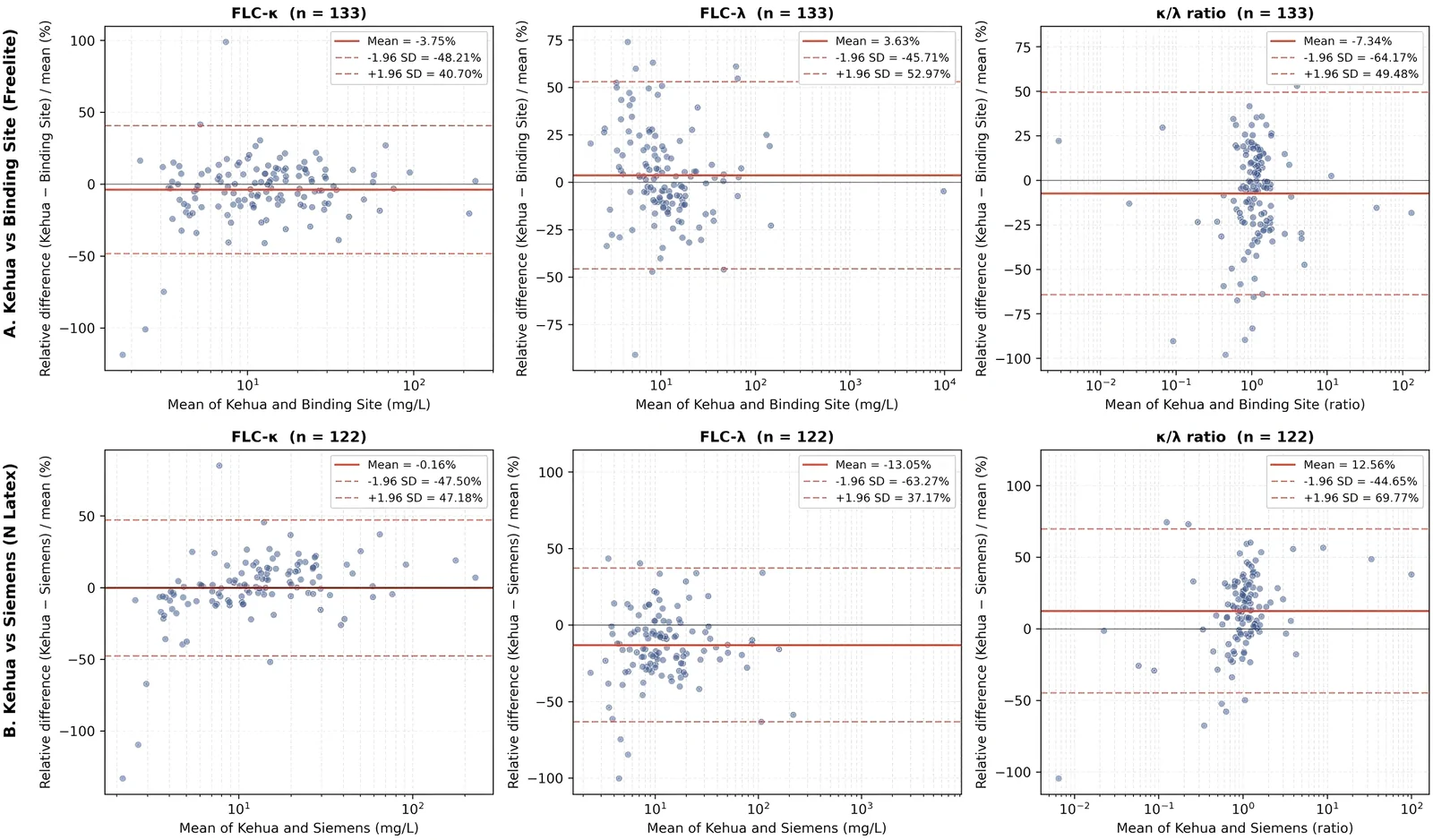
Bland-Altman analysis of relative percentage differences between the Kehua assay and the two imported methods, plotted against the mean concentration on a logarithmic scale. The solid red line indicates the mean bias; dashed red lines indicate the 95% limits of agreement (mean ± 1.96 SD).

### 3.4 Qualitative concordance between systems

The diagnostic classification (positive or negative for each analyte) using method-specific reference intervals is summarised in **Table 3**. Against Freelite, total concordance was 93.23% (124/133) for FLC-κ, 90.98% (121/133) for FLC-λ and 89.47% (119/133) for the κ/λ ratio, corresponding to discordant counts of 9/133, 12/133 and 14/133, respectively. The corresponding Cohen’s kappa coefficients were 0.848 (95% CI 0.752-0.944), 0.799 (0.691-0.908) and 0.675 (0.514-0.836), corresponding to almost-perfect, substantial and substantial agreement. Against N Latex, total concordance was 91.80% (112/122) for FLC-κ, 83.61% (102/122) for FLC-λ and 90.16% (110/122) for the κ/λ ratio, corresponding to discordant counts of 10/122, 20/122 and 12/122, with kappa values of 0.836 (0.739-0.933), 0.673 (0.542-0.804) and 0.710 (0.554-0.866). Positive concordance was consistently higher for Kehua vs N Latex than for Kehua vs Freelite across all three analytes (FLC-κ, 96.36% vs 90.91%; FLC-λ, 88.89% vs 79.59%; κ/λ ratio, 95.24% vs 68.97%). Negative concordance, by contrast, was consistently higher for Kehua vs Freelite than for Kehua vs N Latex (FLC-κ, 94.38% vs 88.06%; FLC-λ, 97.62% vs 79.41%; κ/λ ratio, 95.19% vs 89.11%). Thus, discordance was most apparent for the composite κ/λ ratio and for the N Latex FLC-λ comparison, consistent with the known greater inter-method variability of λ measurement and ratio calculation. Importantly, samples reaching the markedly abnormal κ/λ ratio range used for the IMWG ≥100 biomarker were concordantly identified by the three platforms in this cohort (see Discussion); therefore, the residual discordance mainly affects reference-interval abnormality calls and interpretation near method-specific cut-offs rather than obviously extreme monoclonal FLC patterns. Because discordant calls were not adjudicated against an independent diagnostic standard, they are best interpreted as method-dependent classification differences. These figures are comparable to the published concordance between Freelite and N Latex in similar method-comparison studies (8, 22). To make these discrepancies more transparent, **Supplementary Table 1** summarizes the concordant and discordant case counts for each analyte-comparator pair and highlights that the largest discordance burden was observed for the N Latex FLC-λ classification (20/122, 16.39%), whereas κ/λ ratio discordance was 14/133 (10.53%) against Freelite and 12/122 (9.84%) against N Latex.

**Table 3:** Qualitative concordance between the Kehua FLC assay and the two imported reference systems for κ, λ and κ/λ ratio classification.

Comparison	Analyte	n	Positive concordance (%)	Negative concordance (%)	Total concordance (%)	Cohen’s κ (95% CI)
Kehua vs Freelite	FLC-κ	133	90.91	94.38	93.23	0.848 (0.752-0.944)
Kehua vs Freelite	FLC-λ	133	79.59	97.62	90.98	0.799 (0.691-0.908)
Kehua vs Freelite	κ/λ ratio	133	68.97	95.19	89.47	0.675 (0.514-0.836)
Kehua vs N Latex	FLC-κ	122	96.36	88.06	91.80	0.836 (0.739-0.933)
Kehua vs N Latex	FLC-λ	122	88.89	79.41	83.61	0.673 (0.542-0.804)
Kehua vs N Latex	κ/λ ratio	122	95.24	89.11	90.16	0.710 (0.554-0.866)

Reference intervals applied for Kehua: Freelite intervals (κ 3.3-19.4, λ 5.7-26.3, κ/λ 0.26-1.65) for the Kehua-Freelite comparison; N Latex intervals (κ 6.7-22.4, λ 8.3-27.0, κ/λ 0.31-1.56) for the Kehua-N Latex comparison. Kappa interpreted per Landis & Koch.

### 3.5 Comparator-defined classification performance of the Kehua assay

Using each of the two imported methods in turn as the qualitative reference comparator for abnormality (κ elevation, λ elevation, or κ/λ ratio outside the interval in either direction), the comparator-defined classification performance of the Kehua assay was characterised by ROC analysis (**Figure 4** and **Table 4**). This analysis evaluates how well Kehua reproduces the imported assay classification and should not be interpreted as diagnostic accuracy against an independent disease gold standard. For predicting Freelite-defined FLC-κ elevation, the Kehua FLC-κ value gave an AUC of 0.994 with an optimal cut-off of 19.35 mg/L providing 97.6% sensitivity and 98.9% specificity. The corresponding figure for predicting Freelite-defined FLC-λ elevation was AUC 0.996, with an optimal Kehua FLC-λ cut-off of 22.44 mg/L (100% sensitivity, 96.5% specificity). When the Siemens N Latex assay was used as the reference comparator, the AUC values for Kehua FLC-κ and FLC-λ remained extremely high at 0.999 and 0.997, respectively, with optimal cut-offs of 24.68 mg/L (100% sensitivity, 98.2% specificity) and 21.08 mg/L (100% sensitivity, 95.6% specificity). For predicting κ/λ ratio abnormality, the absolute value of log_10_(κ/λ) measured by Kehua gave AUC values of 0.869 versus Freelite and 0.796 versus N Latex, with sensitivity/specificity at the Youden-optimal cut-offs of 82.8%/79.8% and 62.5%/91.1%, respectively. The lower AUC for the κ/λ ratio reflects the recognised greater inter-method variability of this composite biomarker.

**Figure 4:**
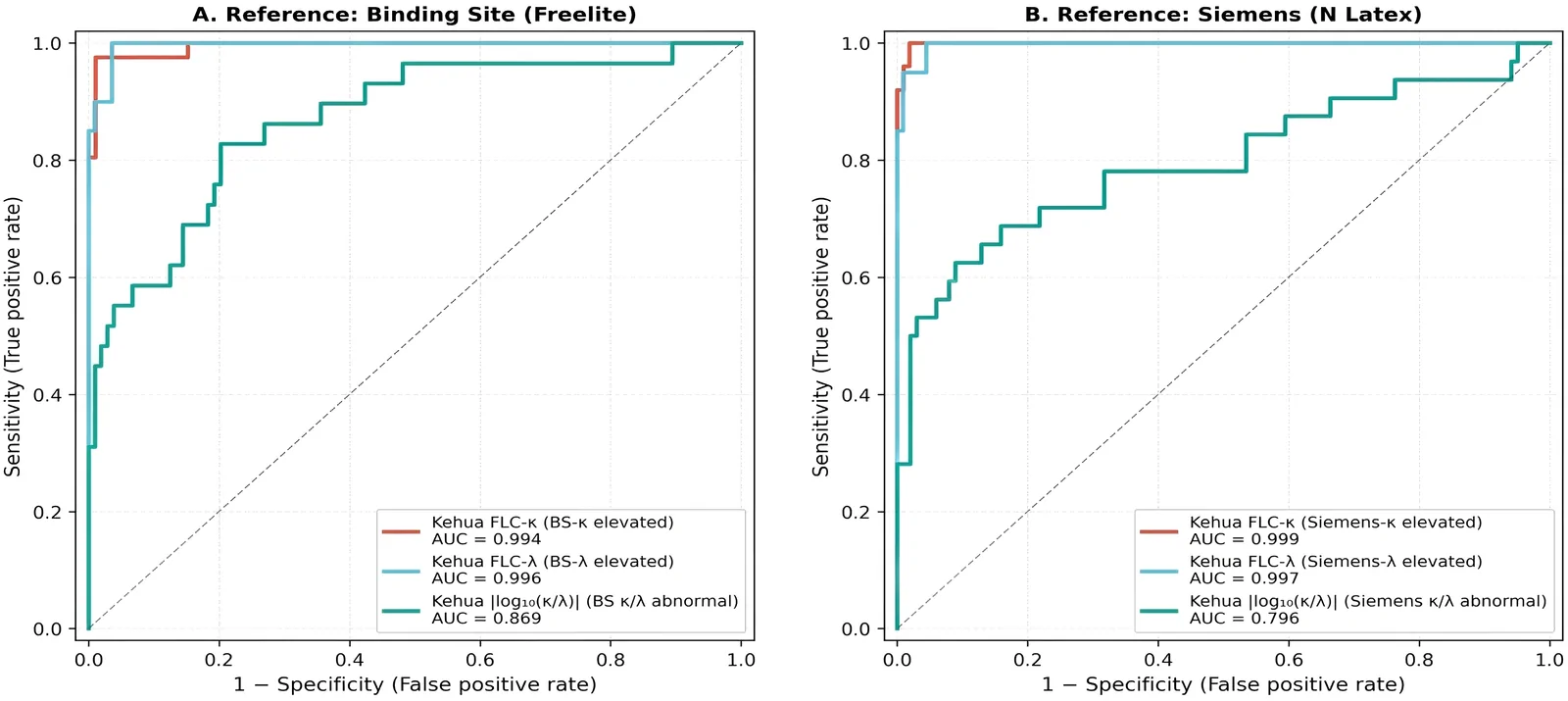
ROC curves for the Kehua FLC assay using the two imported methods in turn as qualitative reference comparators [**(A)** Freelite; **(B)** N Latex]. FLC-κ and FLC-λ used the raw Kehua concentration as the score; the κ/λ ratio used the absolute value of log_10_(κ/λ) to capture deviations in either direction. These curves evaluate concordance with comparator assay classification and not diagnostic accuracy against an independent clinical gold standard.

**Table 4:** Comparator-defined ROC classification performance of the Kehua FLC assay using the two imported assays in turn as qualitative reference comparators.

Reference	Kehua predictor	Reference outcome	AUC	Optimal cut-off	Sens (%)	Spec (%)
Freelite	FLC-κ (mg/L)	FLC-κ elevated	0.994	19.35	97.56	98.91
Freelite	FLC-λ (mg/L)	FLC-λ elevated	0.996	22.44	100.00	96.46
Freelite	|log_10_(κ/λ)|	κ/λ ratio abnormal	0.869	0.190	82.76	79.81
N Latex	FLC-κ (mg/L)	FLC-κ elevated	0.999	24.68	100.00	98.15
N Latex	FLC-λ (mg/L)	FLC-λ elevated	0.997	21.08	100.00	95.58
N Latex	|log_10_(κ/λ)|	κ/λ ratio abnormal	0.796	0.266	62.50	91.09

AUC, area under the ROC curve. Optimal cut-offs determined by Youden’s J statistic. “Elevated” denotes a value above the upper reference limit of the comparator method; “abnormal” denotes a κ/λ ratio outside the reference interval in either direction. These analyses evaluate concordance with comparator assay classification and should not be interpreted as diagnostic accuracy against an independent disease gold standard.

## 4 Discussion

Serum free light chain measurement is firmly established as a key diagnostic and monitoring tool in plasma cell disorders, with the involved/uninvolved κ/λ ratio acting as a myeloma-defining biomarker in current IMWG criteria (1, 2, 6). Until recently, sFLC measurement in Chinese clinical laboratories has depended largely on imported reagents, creating potential challenges for local implementation and supply continuity. The present study evaluated the analytical and clinical agreement of a domestic Kehua latex-enhanced immunoturbidimetric serum sFLC assay run on a Beckman Coulter AU5800 analyser against the two most widely used imported assays-Freelite and N Latex-in a real-world cohort of 133 patients with suspected or established monoclonal gammopathies. The data support three principal conclusions: the Kehua assay demonstrates acceptable within-run precision, it correlates strongly with both imported methods, and it provides good clinical agreement for κ, λ and κ/λ abnormality classification when method-specific intervals are maintained.

With respect to analytical precision, the available within-run CV values (FLC-κ low 1.52-3.88%, high 0.95-2.67% across four runs; FLC-λ low 2.43-3.58%, high 0.67-4.66% across two runs) met the < 10% precision goal recommended by CLSI EP05-A3 and were generally comparable to or better than the published precision of Freelite and N Latex, in which inter-run CVs of 4-7% have been reported in clinical laboratories (9, 14, 23). Importantly, all mean values for both control levels fell within the manufacturer-defined acceptance windows, indicating acceptable short-term calibration stability during the study period. This level of precision is sufficient to support a method-comparison evaluation of the Kehua assay in routine quantification, although the present study cannot speak directly to long-term lot-to-lot reproducibility or to within-laboratory precision across multiple operators and instruments, which would require the more extensive nested design recommended by EP05 (18).

Quantitative agreement with the Freelite assay was excellent, with Pearson correlations of 0.988-1.000, Lin’s concordance correlation coefficients of 0.983-0.999 and Passing-Bablok slopes within 8% of unity for FLC-κ, FLC-λ and κ/λ ratio. The agreement against the N Latex assay was likewise strong (Pearson r 0.991-0.998), but with greater proportional bias-particularly for the κ/λ ratio, where the Passing-Bablok slope of 0.733 means that at very high or very low ratios the Kehua and N Latex absolute values diverge. This proportional bias is not negligible for clinical interpretation because the κ/λ ratio is used in IMWG diagnostic criteria and in smouldering myeloma risk stratification. Consequently, ratio values generated by Kehua and N Latex should be interpreted using method-specific intervals, and longitudinal follow-up should remain on a single platform. This pattern is directionally consistent with the well-recognised differences between the two imported methods themselves, where Freelite typically yields higher FLC-κ and κ/λ values than N Latex, while N Latex generally returns higher FLC-λ values (8, 11). Importantly, the Bland-Altman plots showed no concentration-dependent drift in relative bias over more than four orders of magnitude, suggesting that systematic discrepancy can be approached by method-specific interpretation rather than cross-platform interchangeability.

In addition to quantitative correlation, qualitative agreement is the more clinically relevant outcome for an assay used in screening and risk stratification. The total concordance rates of 83.6-93.2% and Cohen’s kappa values of 0.67-0.85 observed in our study fall well within the range reported for comparisons between Freelite and N Latex themselves, where total concordance has been reported as 80-92% and kappa as 0.65-0.90 in cohorts of patients with monoclonal gammopathies (8–10, 22). The strongest agreement was observed for FLC-κ (kappa 0.85 versus Freelite, 0.84 versus N Latex), in keeping with the well-described observation that κ values are generally more reproducible between platforms than λ values, partly because of the lower frequency of polymerisation artifacts and partly because the dynamic range of κ is narrower (8, 10). The slightly lower kappa for the κ/λ ratio is also consistent with prior comparisons, reflecting the compounding of κ and λ measurement uncertainty in the ratio.

The added discordance summary further distinguishes routine reference-interval classification shifts from high-ratio clinical decision thresholds. The highest discordance burden involved N Latex FLC-λ (20/122, 16.39%), whereas κ/λ ratio discordance remained at approximately 10% in both imported-comparator analyses. This pattern supports cautious cross-platform interpretation of borderline results and reinforces the recommendation that the same assay platform should be used for longitudinal monitoring of an individual patient.

The ROC analysis adds a practical view of classification agreement, but it should not be regarded as evidence of diagnostic interchangeability. When the imported method’s classification was used as the reference comparator, the Kehua assay achieved AUC values approaching 1.0 (0.994-0.999) for reproducing elevated FLC-κ or FLC-λ calls, and AUC values of 0.796–0.869 for reproducing κ/λ ratio abnormality. The Youden-optimal cut-offs derived for the Kehua-Freelite comparison (FLC-κ 19.35 mg/L, FLC-λ 22.44 mg/L) lie close to the published upper reference limits of the Freelite assay itself (19.4 mg/L for κ and 26.3 mg/L for λ) (9, 10). This supports good method concordance for concentration-based elevation calls, but it does not establish diagnostic accuracy against an independent gold standard or justify unrestricted transfer of reference intervals before dedicated reference-interval studies in the target population. The lower AUC for the κ/λ ratio reproduces a well-known limitation: because the ratio is a composite of two assays each affected by its own bias, even small individual biases combine into appreciable variation in the ratio at extreme values (10, 11).

The clinical implications of these findings should be interpreted in the context of contemporary IMWG criteria. The involved/uninvolved sFLC ratio threshold of 100 is a myeloma-defining event when the absolute concentration of the involved light chain is ≥ 100 mg/L (1, 2); in our cohort the Kehua and the two imported methods returned ratios > 100 for the same samples, and the absolute FLC-κ values in those samples were of comparable magnitude across the three platforms (e.g. 237.91, 232.87 and 222.0 mg/L for the most extreme κ-restricted sample). This finding suggests that the Kehua assay can identify markedly abnormal serum FLC patterns, but it does not remove the need for method-specific interpretation near diagnostic thresholds. As has been pointed out for other domestic and third-party assays, the originally derived threshold of 100 must be interpreted in the light of the assay used to derive it, and the same platform should be used to monitor a given patient over time (10, 11, 24, 25). The same caveat applies to the high-risk smouldering MM 2/20/20 rule, which uses an sFLC ratio of 20 as one criterion (2, 7).

The potential implementation implications of a domestic sFLC assay should also be interpreted conservatively. The MM disease burden in China is rising rapidly (3, 4, 26), and population-level screening programmes such as the iStopMM study have demonstrated that the prevalence of subclinical light-chain MGUS far exceeds previous estimates, particularly when sensitive sFLC assays are used (27, 28). A serum sFLC test compatible with routine biochemistry analysers could in principle support broader laboratory access, primary-care evaluation of monoclonal gammopathies and serial monitoring of patients with AL amyloidosis and oligosecretory disease (29, 30). However, the present study did not include reagent-cost analysis, distribution assessment, reimbursement evaluation, workflow-timing data or laboratory implementation modelling. Therefore, statements regarding affordability, availability and screening expansion should be understood as plausible implementation advantages requiring separate health-economic and operational validation, not as direct outcomes demonstrated by this dataset. It should also be emphasised that, just as Freelite and N Latex are not interchangeable (8, 11), the Kehua assay is not an exact replacement for either imported method, and clinical and laboratory teams should commit to a single assay for the longitudinal follow-up of an individual patient.

This study has several limitations. First, it is a single-centre evaluation with a moderate sample size (n = 133); larger multi-centre studies covering a wider patient and disease spectrum will be necessary to confirm performance across regions and reagent lots. Second, although our cohort included a range of plasma cell disorders, it was enriched for established disease and may not fully reflect screening-phase populations. Third, the IMWG and AL amyloidosis treatment-response criteria are derived from Freelite data; whether identical thresholds will perform identically for clinical decision making with the Kehua assay requires prospective outcome-based validation. Fourth, this was a method-comparison study rather than a complete analytical-validation study. Limits of blank, detection and quantification, carry-over, lot-to-lot reproducibility, extended post-dilution linearity, antigen-excess behaviour and major interference studies beyond routine exclusion of gross haemolysis, lipaemia and icterus were not fully assessed. Fifth, only serum samples were evaluated; the findings cannot be extrapolated to urine or cerebrospinal fluid, and urine FLC testing remains relevant in the broader laboratory evaluation of monoclonal gammopathies. Sixth, the product information available for the evaluated Kehua lots did not disclose the clonality or production route of the immobilized anti-free-light-chain antibodies, limiting mechanistic interpretation of any potential epitope-related assay differences. Seventh, discordant qualitative calls were analysed at the method-comparison level and were not adjudicated against an independent clinical gold standard or outcome endpoint. Finally, although our qualitative analysis used method-specific reference intervals, dedicated reference-interval studies in a Chinese healthy population, ideally accounting for age and renal function as recommended by recent iStopMM-derived work (27, 28), will be needed to optimise the Kehua-specific cut-offs.

## 5 Conclusion

The Kehua latex-enhanced immunoturbidimetric serum free light chain assay run on the Beckman Coulter AU5800 analyser shows acceptable within-run precision (CV < 5% at both control levels), strong quantitative correlation (Pearson r > 0.99 for FLC-κ, FLC-λ and κ/λ ratio against both Freelite and N Latex), and substantial-to-almost-perfect qualitative agreement (Cohen’s κ 0.67-0.85) with two long-established imported systems in a real-world cohort of patients with plasma cell disorders. Comparator-defined ROC analysis shows that the Kehua assay closely reproduces the imported methods’ classification of FLC-κ and FLC-λ elevation and provides moderate-to-good concordance for κ/λ ratio abnormality. The Kehua serum assay can therefore be considered a viable domestic option for routine serum sFLC quantification, provided that method-specific reference intervals are retained and that serial monitoring of an individual patient uses one and the same platform. Multi-centre validation, complete analytical validation, dedicated Chinese reference-interval studies, urine-matrix evaluation and outcome-based comparison against IMWG decision thresholds remain priorities for future work.

## Data Availability

The original contributions presented in the study are included in the article/**Supplementary Material**. Further inquiries can be directed to the corresponding authors.
