# Supplementary material for: Consistency and correlation of a domestic Kehua serum free light chain assay with two imported analytical systems
Source: Front Oncol. 2026 Oct 15;16:1900671. doi: 10.3389/fonc.2026.1900671 (PMC13716613; doi:10.3389/fonc.2026.1900671)
Supplement: Supplementary file 1 [file Table1.docx]

**Supplementary Table S1**

**Summary of qualitative discordance between the Kehua FLC assay and imported comparator systems**

| **Comparator** | **Classification target** | **Evaluable n** | **Concordant n (%)** | **Discordant n (%)** | **Interpretation** |
| --- | --- | --- | --- | --- | --- |
| Kehua vs Freelite | FLC-κ elevation | 133 | 124 (93.23) | 9 (6.77) | Limited qualitative mismatch; quantitative agreement remained strong. |
| Kehua vs Freelite | FLC-λ elevation | 133 | 121 (90.98) | 12 (9.02) | More discordance than FLC-κ, consistent with greater inter-method variability of λ measurement. |
| Kehua vs Freelite | κ/λ ratio abnormality | 133 | 119 (89.47) | 14 (10.53) | Composite-ratio discordance mainly affects reference-interval abnormality calls near method-specific cut-offs. |
| Kehua vs N Latex | FLC-κ elevation | 122 | 112 (91.80) | 10 (8.20) | Limited qualitative mismatch under N Latex reference intervals. |
| Kehua vs N Latex | FLC-λ elevation | 122 | 102 (83.61) | 20 (16.39) | Largest discordance burden, supporting cautious interpretation of λ values across platforms. |
| Kehua vs N Latex | κ/λ ratio abnormality | 122 | 110 (90.16) | 12 (9.84) | Composite-ratio discordance remained below 10% but supports same-method longitudinal monitoring. |

Note. FLC = free light chain. Values are derived from the qualitative concordance results in Table 3 of the revised manuscript. Discordance was calculated as evaluable n minus total concordant cases and summarizes aggregate method-dependent classification mismatch; discordant calls were not adjudicated against an independent diagnostic gold standard or outcome endpoint. In this cohort, markedly abnormal κ/λ ratio results in the IMWG ≥100 range were concordantly identified across platforms, whereas residual discordance mainly concerned reference-interval abnormality calls near method-specific cut-offs.
